# Identification and analysis of KLF13 variants in patients with congenital heart disease

**DOI:** 10.1186/s12881-020-01009-x

**Published:** 2020-04-15

**Authors:** Wenjuan Li, Baolei Li, Tingting Li, Ergeng Zhang, Qingjie Wang, Sun Chen, Kun Sun

**Affiliations:** Department of Pediatric Cardiology, Xinhua Hospital, Affiliated to Shanghai Jiao Tong University School of Medicine, 1665 Kongjiang Road, Shanghai, 200092 China

## Abstract

**Background:**

The protein Kruppel-like factor 13 (KLF13) is a member of the KLF family and has been identified as a cardiac transcription factor that is involved in heart development. However, the relationship between KLF13 variants and CHDs in humans remains largely unknown. The present study aimed to screen the KLF13 variants in CHD patients and genetically analyze the functions of these variants.

**Methods:**

KLF13 variants were sequenced in a cohort of 309 CHD patients and population-matched healthy controls (*n* = 200) using targeted sequencing. To investigate the effect of variants on the functional properties of the KLF13 protein, the expression and subcellular localization of the protein, as well as the transcriptional activities of downstream genes and physical interactions with other transcription factors, were assessed.

**Results:**

Two heterozygous variants, c.487C > T (P163S) and c.467G > A (S156N), were identified in two out of 309 CHD patients with tricuspid valve atresia and transposition of the great arteries, respectively. No variants were found among healthy controls. The variant c.467G > A (S156N) had increased protein expression and enhanced functionality compared with the wild type, without affecting the subcellular localization. The other variant, c.487C > T (P163S), did not show any abnormalities in protein expression or subcellular localization; however, it inhibited the transcriptional activities of downstream target genes and physically interacted with TBX5, another cardiac transcription factor.

**Conclusion:**

Our results show that the S156N and P163S variants may affect the transcriptional function of KLF13 and physical interaction with TBX5. These results identified KLF13 as a potential genetic risk factor for congenital heart disease.

## Background

Congenital heart disease (CHD) is a term that refers to any anatomical defect in the heart or major blood vessels that is present at birth. CHD affects 1% of newborn babies, and approximately one to two per 1000 newborn babies have critical CHDs that can cause death during the neonatal period, such as pulmonary atresia and transposition of the great arteries [1–3]. Infants and children with CHDs often suffer from delays in growth and serious progressive cardiac complications [4]. Despite significant advances in our understanding of cardiac morphogenesis, emerging evidence suggests that genetic risk factors play a crucial role in the etiology of CHD. Further elucidation of the molecular and genetic intricacies of CHDs will likely be an important step in improving treatments for CHD patients.

The heart is the first functional organ to develop during vertebrate embryogenesis, which is a complex process in which multiple cell lineages contribute to organ development. Epithelial–mesenchymal transformation (EMT) is an important step during heart development and is regulated by myocardial signals to generate mesenchymal cells that migrate into the cardiac jelly and proliferate to cellularize the endocardial cushions [5, 6]. Malformation of the atrioventricular canal cushions can lead to atrioventricular septal defects (AVSDs) or other endocardial cushion defects. Kruppel-like factor 13 (KLF13) protein has been identified as a novel collaborator of the cardiac GATA4 and TBX5 factors, which are highly expressed in the endocardium and myocardium during embryonic development. Knocking down KLF13 alleles in Xenopus embryos leads to valve immaturity, septal defects, and hypotrabeculation [7–9]. However, the mechanisms of KLF13 dysfunction causing CHDs are still poorly understood.

A spatiotemporal expression analysis of KLF13 in murine hearts showed that the earliest sign of expression was at E9.5, and subsequent expression was detected in the developing atrial myocardium, ventricular trabeculae, AV cushions, and truncus arteriosus, which then decreased in the postnatal heart [7]. Additionally, in *Xenopus* embryos, the highest levels of KLF13 were found in the interventricular septum and the atrioventricular valves, and knocking down KLF13 induced ventricular hypotrabeculation, atrial septal defects (ASDs) and delayed AV cushion formation [ [7]].

KLF13 is a member of the KLF family of transcription factors that have been categorized along with the specificity protein (Sp) family of transcription factors [10]. To date, twenty-one KLF members have been identified in humans. These transcription factors are characterized by C-terminal Cys_2_His_2_ (C_2_H_2_) zinc-finger motifs that confer preferential binding to GC/GT rich sequences in gene promoters and enhancer regions to activate or inhibit downstream target expression [11, 12]. ANF and BNP are natriuretic peptides that are expressed in the heart and are developmentally regulated. Their levels rise continuously during embryonic cardiac development in both atria and ventricles when cells differentiate into cardiomyocytes. Their levels then drop after postnatal development. At the genetic level, analysis of the ANP and BNP promoters has led to the characterization of key cardiac transcription factors that govern cardiac growth and differentiation [13]. Studies by Lavallee et al. revealed that KLF13 could activate the BNP promoter [ [7]].

Additionally, analysis of the binding interactions of transcription factor variants helps us better understand how the disruption of combinatorial interactions can lead to specific congenital disabilities. TBX5 is a member of the T-box transcription factor family which has crucial roles in regulating early cellular commitment, differentiation, and heart development [14]. TBX5 cooperates with other transcription factors, such as GATA4 and NKX2.5, to synergistically regulate downstream targets during cardiac development [ [15, 16]]. Previously, we showed that KLF13 is a TBX5 cofactor and that KLF13 may be a genetic modifier of Holt-Oram Syndrome and possibly other congenital heart diseases [9].

However, since current studies of KLF13 have focused on animal models [ [7, 17]], it remains unclear whether genetic variants are involved in the mechanisms of CHDs in humans. In the present study, we identified two KLF13 heterozygous variants in a cohort of patients with complex CHDs and compared them with those of healthy controls to evaluate the prevalence of KLF13 variants in sporadic CHDs. Our results demonstrated that these variants altered protein expression, changed the transcriptional activation of BNP and impaired the genetic interaction of KLF13 with TBX5.

## Methods

### Study subjects

In this study, we recruited a total of 309 patients with complex CHDs. These patients were diagnosed by echocardiography or cardiac catheterization or underwent cardiac surgery at Shanghai Xinhua Hospital. The patients included 191 males and 118 females (Table 1). Patients with known syndromic CHDs or chromosomal abnormalities were excluded from our study. The controls were 200 population-matched healthy children without heart disease. The study protocol was reviewed and approved by the Xinhua Hospital Ethics Committee. Both parents and legal guardians of the patients and healthy controls provided signed informed consent. Subsequently, peripheral blood was collected for DNA extraction. Genomic DNA was extracted using the QIAamp DNA Blood Mini Kit (Qiagen, Germany) and stored at − 80 °C.

**Table 1:** Clinical information of the 309 CHDs patients

Parameter	Range or number	Mean value or percentage
Female	118	38.19%
Male	191	61.81%
Age	10d-9y	/
**Types of CHDs**		
TOF	98	31.72%
DORV	59	19.09%
TGA	50	16.18%
PA	62	20.06%
TA	10	3.24%
IAA	9	2.91%
SV	21	6.80%

**Abbreviations**: *TOF* Tetralogy of Fallot; *DORV* Double outlet right ventricle; *TGA* Transposition of the great arteries; *PA* Pulmonary atresia; *TA* Tricuspid; valve atresia; *IAA* Interrupted aortic arch; *SV* Single ventricle

### Target sequencing and analysis

Genomic DNA was sequenced by target sequencing technology using the Illumina HiSeq 2000 platform for variants of KLF13 (GenBank accession number NC_000015.10, NM_015995.3) and several other cardiac transcriptional factors involved in cardiovascular development (GATA4, TBX5, TBX1, GATA6, GATA5 and so on). Then, Sanger sequencing was performed to validate all the candidate variants. To evaluate the protein characteristics of nonsynonymous variants, we used SIFT (http://sift.jcvi.org/), PolyPhen-2 (http://genetics.bwh.harvard.edu/pph2/), and Mutation Taster (www.mutationtaster.org/). Amino acid substitutions were predicted as damaging when the score was ≤0.05 in SIFT or ≥ 0.85 in PolyPhen-2. KLF13 protein sequences from *Homo sapiens* (human), *Mus musculus* (house mouse), *Bos taurus* (cattle), *Capra hircus* (goat), *Pan troglodytes* (chimpanzee), *Xenopus laevis* (frog) and *Sus scrofa* (swine) were downloaded from the Universal Protein (UniProt) database (http://uniprot.org/) and aligned with Clustal X software.

### Plasmid construction and site-directed mutagenesis

The KLF13 and TBX5 cDNA plasmids were purchased from Genomeditech. Site-directed mutagenesis for the KLF13 point mutations c.467G > A (S156N) and c.487C > T (P163S) was performed according to the protocol provided for the Site-Directed Mutagenesis Kit (Stratagene, USA). Then, the mutated sites were confirmed with Sanger sequencing. The luciferase reporter with human B-type natriuretic peptide (BNP) promoter was constructed as previously described [ [18]].

### Cell cultures and transfection

For cell culture experiments, 293 T cells were used for protein extraction and immunofluorescent staining. NIH 3 T3 cells were used for luciferase assays and were maintained in growth medium (Dulbecco’s modified Eagle’s medium) supplemented with 10% fetal bovine serum and 1% penicillin/streptomycin. Plasmids were transfected with FuGene HD (Promega, USA) according to the manufacturer’s protocol 24 h after the cells were seeded.

### Luciferase assays

NIH 3 T3 cells were seeded onto a 24-well plate, and 600 ng of BNP luciferase reporter vector, different dosages (25, 50, 100, 200, and 300 ng) of the wild-type/variant KLF13 plasmid, and pCMV-Tag2B vector with or without TBX5 plasmids were cotransfected with FuGene HD. The luciferase activity was measured by the Dual-Glo Luciferase Assay System (Promega) following the manufacturer’s protocol after 48 h of transfection. Firefly luciferase activities are reported as fold activation. All experiments were repeated at least three times in duplicate.

### Immunofluorescence

For myc staining, 293 T cells were harvested 48 h after transfection with wild-type KLF13 or its variants. After fixation with 4% paraformaldehyde at room temperature for 10 min, permeabilization with 0.3% Triton X-100 for 15 min, and blocking with 5% BSA for 1 h, cells were incubated with the primary antibody (1:500, rabbit polyclonal, anti-myc, 16,286–1-AP, Proteintech, USA) overnight at a temperature of 4 °C, followed by incubation with the conjugated secondary antibody, anti-rabbit Alexa Fluor® 488 (1:500, R37116, Life Technologies, USA) at room temperature in the dark for 1 h. DAPI (Vector Laboratories, USA) was used for nuclear staining. The images were acquired using fluorescence microscopy at 200X magnification with Image-Pro Plus software.

### Western blotting analysis

293 T cells were harvested at 48 h after transfection with wild-type KLF13 or its variants. The protein was extracted as previously described [14]. A total of 50 μg of the extract was subjected to 10% SDS-PAGE, transferred onto nitrocellulose membranes and blocked with skim milk (5%) in TBST (0.1%) at room temperature for 2 h, using gentle agitation during the process. Then, the membranes were incubated with the primary antibody (myc: 1:1000, rabbit polyclonal anti-myc, 16,286–1-AP, Proteintech, USA; GAPDH: 1:1000, rabbit monoclonal anti-GAPDH, ab181602, Abcam, USA) overnight at a temperature of 4 °C. Following incubation with the primary antibody, horseradish peroxidase-conjugated secondary antibody (1:1000, 111–005-003, Jackson ImmunoResearch, USA) and Immobilon Western Chemiluminescent HRP Substrate (Millipore, USA) were used to visualize chemiluminescent immunodetection.

### Coimmunoprecipitation

293 T cells were transfected with wild-type KLF13 or variants and TBX5 plasmids using FuGene HD according to the manufacturer’s guidelines. Protein extracts were incubated overnight with anti-myc antibody (1:100, rabbit polyclonal anti-myc, 16,286–1-AP, Proteintech, USA) coupled with magnetic beads. Bound proteins were revealed with anti-TBX5 (1:1000, rabbit polyclonal anti-TBX5, 42–6500, Invitrogen, USA) antibody by Western blotting.

### Statistical analysis

Data are reported as the means ± SEMs. Two-way analysis of variance (ANOVA) was used to compare differences among groups by SPSS. A *P* value < 0.05 was selected to indicate statistical significance.

## Results

### KLF13 sequencing in CHD patients

**Fig. 1:**
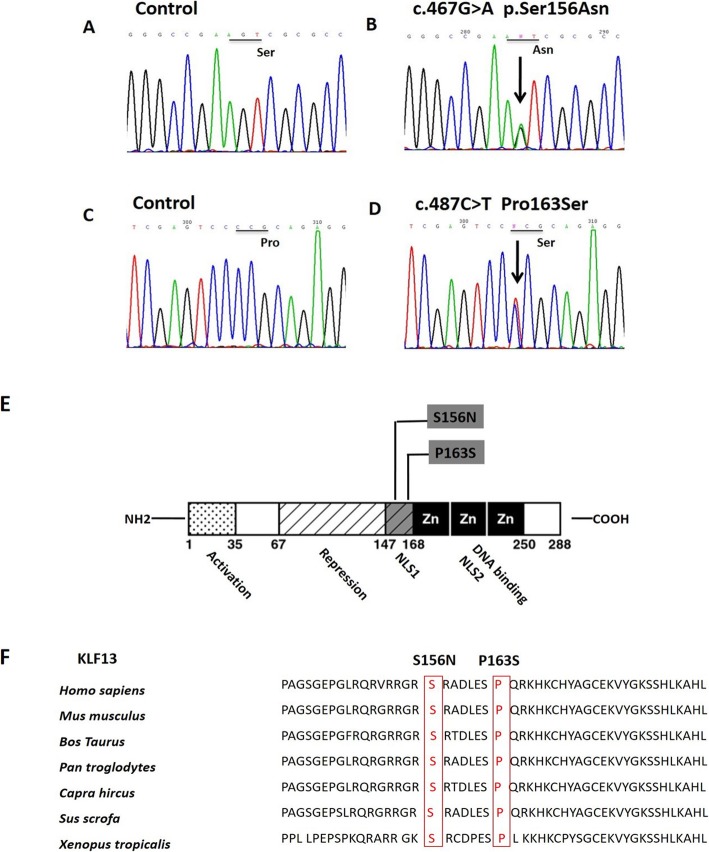
Sequence chromatograms of KLF13 missense variants in patients and controls. **a** and **c**: Chromatograms of normal controls. **b** and **d**: Chromatograms of the three heterozygous variants. Arrows show heterozygous nucleotide changes; **e**: Location of the KLF13 variants (highlighted in bold). NLS, nuclear-localization signals; Zn, zinc finger domain. **f**: Alignments of KLF13 protein among different species indicated that S156N and P163S were conserved among vertebrates

In this study, a cohort of 309 patients with CHDs was established. Additionally, a total of 200 ethnically matched healthy subjects were enrolled and used as controls. The baseline clinical characteristics of the 309 CHD patients are summarized in Table 1. The entire coding region of the KLF13 gene was sequenced in all patients with CHDs by targeted sequencing. We identified two heterozygous variants of KLF13 in two out of 309 patients. A transversion of proline into serine at amino acid position 163 (exon 1 c.487C > T P163S) was identified in one patient with TA VSD ASD. Additionally, a transversion of serine into asparagine at the first nucleotide of codon 156 of the KLF13 gene (exon 1: c.467G > A S156N) was found in the D-TGA patient (Fig. 1a-d). These two patients did not have any variants in other sequenced genes, and no variants of any kind were found among the control individuals. SIFT, PolyPhen-2, and Mutation Taster were used to perform bioinformatic predictions for these two variants. Details of the specific sequence variants are summarized in Table 2.

**Table 2:** Clinical and baseline characteristics of novel KLF13 variants in CHDs patients

Patient	Age	Cardiac Phenotype	Location in Gene	Function	Nucleotide Change	Amino acid Change	SIFT	Mutation taster	PolyPhen- V2	Allele Frequency
1	7 Months	D-TGA	Exon 1	Missense	c.467G > A	S156N	0.04	0.01652	0.818	0.003236
2	9 Months	TA VSD ASD	Exon 1	Missense	c.487C > T	P163S	0.57	0.93386	0.314	0.003236

### In silico analyses of KLF13 mutant protein

Human KLF13 has 6825 bp on chromosome 15, is composed of two exons and two introns and encodes 288 amino acids. Song et al. analyzed the KLF13 protein structure and revealed that it is composed of three zinc-finger domains (Zns), distinct transcriptional activation and repression domains, and two potent independent nuclear localization signals (NLS) [11]. Both S156N and P163S were located in the NLS1 region that was close to the DNA-binding domain (Fig. 1e). Multiple sequence alignments revealed that the S156N and P163S variants were highly conserved in vertebrates (Fig. 1f), indicating that these variants are important and might result in alteration of gene function.

### Subcellular localization and expression of KLF13 variants

**Fig. 2:**
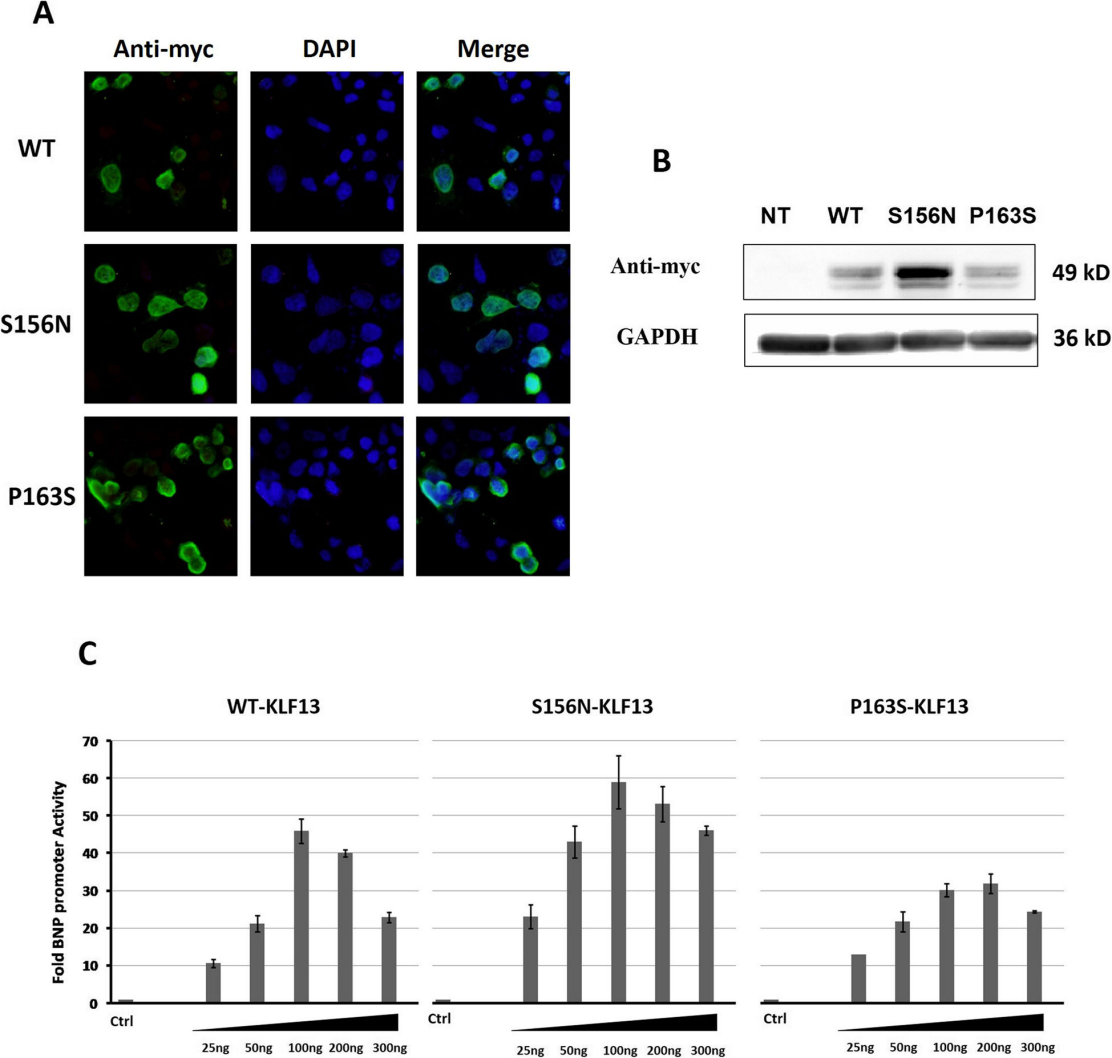
Characterization of KLF13 variants. **a**: Immunocytochemical staining to detect the cellular localization of KLF13 variants transfected in 293 T cells. The green color shows the Alexa 488 conjugated secondary antibodies against the anti-myc antibody. The nuclei were visualized with DAPI. **b**: Western blotting showing the expression of the KLF13 variants. **c**: The transcriptional activities of KLF13 variants on BNP promoter. Transfections were carried out using the human B-type natriuretic peptide (BNP) promoter and increasing dosages (25, 50, 100, 200, and 300 ng) of expression vectors. Each dosage was conducted in duplicate and the experiment was conducted twice

We analyzed the protein expression of these two variants in the nucleus and cytoplasm. The subcellular localization was determined via immunofluorescence (Fig. 2a). Although both mutations were located in the NLS1 region, the variants had an equal protein distribution in the two cellular compartments compared to the wild type. However, measurements of the expression of the myc-tagged-KLF13 variant using total protein extracts prepared from transfected 293 T cells (Fig. 2b) showed that the expression of the S156N variant was distinctly higher than that of wild-type KLF13 (*P* < 0.05), while the P163S variant had a similar expression to that of the wild type, which suggested that the S156N variant might be a gain-of-function variant.

### Transcriptional activity of KLF13 variant protein

To evaluate the functional ability of the KLF13 variants to regulate downstream genes, the transcriptional activities of the variants were analyzed using a *BNP*-luciferase reporter, and cells were cotransfected with wild type or KLF13 variants. As shown in Fig. 2c, a dose response study (25, 50, 100, 200, and 300 ng) showed that, in comparison to wild-type KLF13, the P163S variant had decreased transcriptional activity. In contrast, the S156N variant had increased transcriptional activity, which indicated that the KLF13 variants affected *BNP* promoter transactivation (*P* < 0.001). Therefore, the variants S156N and P163S may affect KLF13 downstream target gene expression during cardiogenesis, for which variant S156N was a gain-of-function mutant, while variant P163S disrupted the function of KLF13.

### KLF13 variants affect the physical interactions with TBX5

**Fig. 3:**
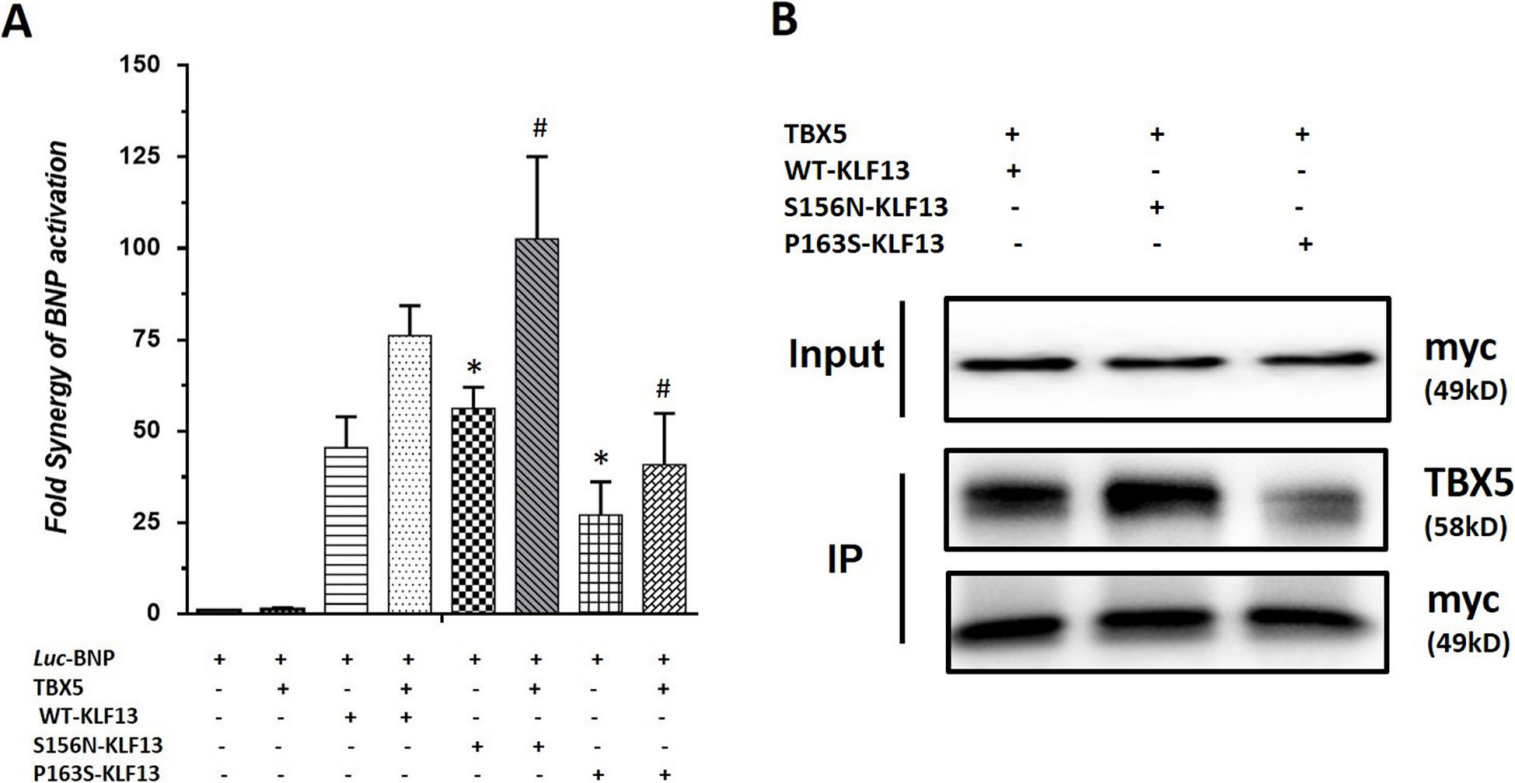
KLF13 variants have effects on genetic interaction with TBX5. **a**: Luciferase activity in NIH3T3 cells cotransfected with wild-type KLF13 or mutant-expression constructs and the *BNP* promoter, showing that the P163S mutant could decrease the synergistic activation of the *BNP* promoter, while the S156N mutant increased the synergistic activation of *BNP*.**b**: Coimmunoprecipitations were carried out using 293 T cells expressing Myc-KLF13 and TBX5 proteins in combination. The top panel shows Western blots of the nuclear extracts used, with the indicated antibodies. The bottom panel is a Western blot of the anti-TBX5 immunoprecipitates. (*p < 0.05, Statistical significant vs WT, # *p* < 0.05, Statistical significant vs WT co-transfected with TBX5)

Previously, we reported that KLF13 is a TBX5 cofactor; these two proteins were found to colocalize in several cardiac cells, and they physically and functionally interact to synergistically activate cardiac promoters [9]. Therefore, to verify the impact of KLF13 variants on physical interactions with TBX5, wild-type or variant KLF13 constructs were cotransfected with TBX5 using the BNP-luciferase reporter. As shown in Fig. 3a, when NIH 3 T3 cells were cotransfected with KLF13 and TBX5, the BNP-luciferase reporter was activated up to 75-fold. Decreased synergistic transcriptional activation was detected with variant P163S, and increased synergy was observed with variant S156N. To test whether the altered synergy was due to altered physical interactions, the binding of wild-type KLF13 or KLF13 variants with TBX5 was tested by co-IP. Figure 3b shows the analysis of the co-IP assay data. P163S showed reduced synergy with TBX5 and had a decreased physical interaction with the TBX5 protein. In contrast, S156N had a significantly increased ability to physically and functionally interact with TBX5.

## Discussion

KLF13, a member of the KLF family of transcription factors, has been identified as a cardiac factor that is involved in heart development [7, 17, 19, 20]. Previous research by Lavallee et al. showed that KLF13 is expressed during cardiac morphogenesis in mice and that knocking it down induced ventricular hypotrabeculation, atrial septal defects (ASDs), and delayed AV cushion formation in Xenopus embryos [7]. However, variants of KLF13 in CHD patients have remained unidentified and uncharacterized. In the present study, two heterozygous KLF13 variants were discovered using targeted sequencing in 309 patients with complex CHDs. Variant c.467G > A (S156N), identified in patients with TA, VSD, and ASD, had altered protein expression and functionality compared with the wild-type protein, without affecting the subcellular localization. The other variant, c.487C > T (P163S), found in a D-TGA patient, did not show any abnormalities in protein expression or subcellular localization. However, this variant had altered transcriptional activities of downstream genes and altered physical interactions with another cardiac factor, TBX5.

Both variants, c.467G > A and c.487C > T, were highly preserved in vertebrates based on multiple sequence alignment analyses and were located in the NLS1 region. Song et al. identified a bipartite NLS in the KLF13 protein based on sequence searching and found that AA 147–168 (NLS1) was not the only NLS region for protein subcellular localization because deletion of this signal did not affect nuclear transport [11]. In our present results, although the changed sequences of these two variants were located in the NLS1 region, the variants were also expressed in the nucleus, as was the wild-type KLF13 protein. However, the locations of the mutations in these two variants were close to the Cys_2_/His_2_ zinc-finger motifs that bind to gene promoters and enhancer regions to activate or inhibit transcription [21], suggesting that these two variants might have crucial functions in mediating the expression of downstream target genes. Lavallee et al. used the BNP promoter and showed that KLF13 could activate the promoter by binding to an evolutionarily conserved CACCC box [7]. Although the subcellular localization of the variant protein did not change, which is in contrast to most previously reported CHD-associated variants [22, 23], we found that the S156N variant was a gain-of-function mutation. The mutation at this site may promote DNA binding activity by increasing the protein levels, which may explain the upregulated BNP in this study. Although a gain-of-function mutation was detected in our experiments, the protein that might be less stable in vivo could also result in a loss-of-function phenotype [24, 25]. Consistent with this observation, human heterozygous microdeletions and duplication of the chromosomal band harboring *KLF13* (15q13.3) have been demonstrated to be associated with a wide range of cardiac defects [26]. Unlike the S156N variant, the c.487C > T variant decreased the transcriptional activity of BNP in the present study, but we did not find any disruption of expression or subcellular localization. However, a proline-to-serine substitution may result in impaired interactions with DNA, and the location of the P163S variant in the tertiary structure that is near the bound DNA might also lead to a decrease in the DNA-binding activity.

The reduced binding ability of our identified variants to their targets may be primarily related to decreased DNA binding. However, structural instability of the variants when bound to cofactors is another possibility [27]. The mechanism of the function of cardiac transcription factors in the developing heart involves cooperative interactions with other conserved factors, and any structural defect is often linked to more than one candidate gene. Additionally, variable expressivity of the phenotype is often observed in cases where CHDs are linked to a specific gene mutation [28]. Thus, variants of these factors may eliminate any cooperative interactions between these cofactors [ [28, 29]]. Previously, we showed that KLF13 and TBX5 exhibit physical and functional interactions and that a combined heterozygous loss of TBX5 and its associated KLF13 leads to decreased postnatal viability and a higher incidence of septal defects compared to that of *Tbx5* heterozygous mice. Additionally, several TBX5 mutations have been associated with CHDs, as indicated by impaired functional and physical interactions with KLF13, which confirms that TBX5 is a genetic cofactor of KLF13 [9]. In the present study, the increased transcriptional activity of *BNP* was more pronounced when wild-type KLF13 was cotransfected with TBX5, which indicated that KLF13 has functional interactions with TBX5 to activate BNP promoters. In our previous study of the structure-function of TBX5-KLF13 synergy [9], the N-terminal domain of KLF13 was mainly found to support synergy with TBX5. The two variants of KLF13 that were detected in the 309 CHD patients were in the N-terminal domain of the protein. However, the two variants had different effects on the interactions with TBX5. Significantly enhanced synergistic activity was detected in the S156N variant, while the P163S variant eliminated this synergistic activity, which suggested that the loss of function observed in KLF13 was associated with disruption of the physical and functional interactions with cofactors (TBX5), while the gain of function could enhance the interactions with cofactors.

This study has some limitations. Corrections between genotype and phenotype remain challenging in exploring CHDs. In this study, although the variant P163S eliminated synergistic activity with TBX5, the patient did not have typical Holt-Oram Syndrome or other TBX5-related features. It is uncertain what other consequences have not been anticipated, such as other transcription factors not investigated in this study that interacted with KLF13. Thus, larger cohort retrospective and prospective studies of CHDs are still needed to pave the way for future elucidation of its etiology.

## Conclusions

This study suggested that CHD patients harbored KLF13 gene variants, and KLF13 variants may contribute to the occurrence of congenital heart disease. Additional studies that test a larger population will help further our understanding of the role of KLF13 in CHD molecular pathogenesis.

## Data Availability

The datasets used and analyzed during the current study are available from the corresponding author on reasonable request.

## Abbreviations

CHD: Congenital heart disease
KLF13: Kruppel-like factor 13
EMT: Epithelial–mesenchymal transformation
AVSD: Atrioventricular septal defects
ASD: Atrial septal defects
VSD: Ventricular septal defects
TA: Tricuspid valve atresia transposition of the great arteries,
TGA: Transposition of the great arteries
NLS: Nuclear localization signals
Zns: Zinc fingers domain
